# Supplementary figures and images for: The conserved coordination of acyl-homoserine lactone and PqsE signaling defines the RhlR-dependent quorum-sensing network in Pseudomonas aeruginosa clinical isolates
Source: bioRxiv. 2026 May 21:2026.05.21.726854. Preprint. [Version 1] doi: 10.64898/2026.05.21.726854 (PMC13228606; doi:10.64898/2026.05.21.726854)

Figure S1

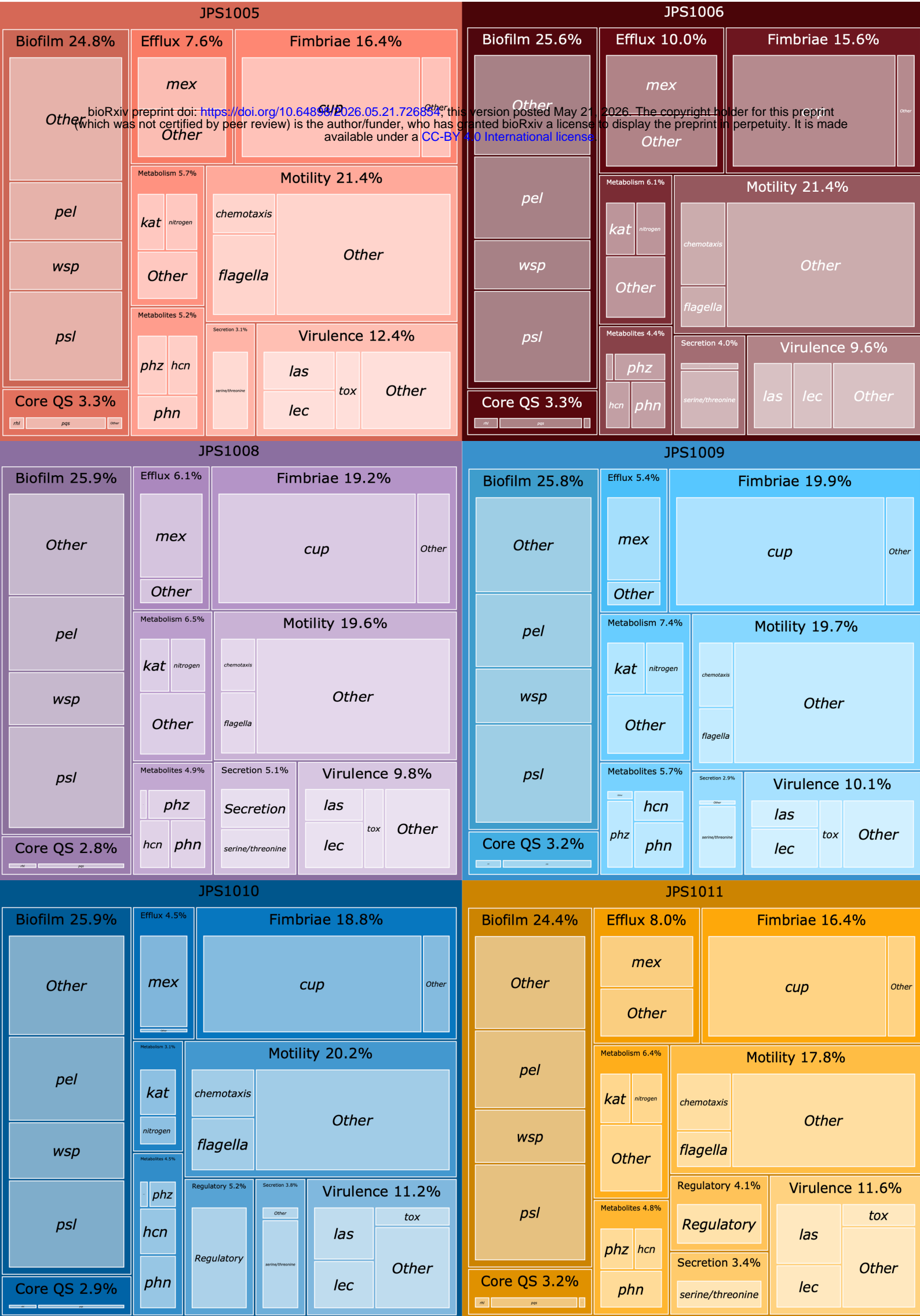

Figure S2

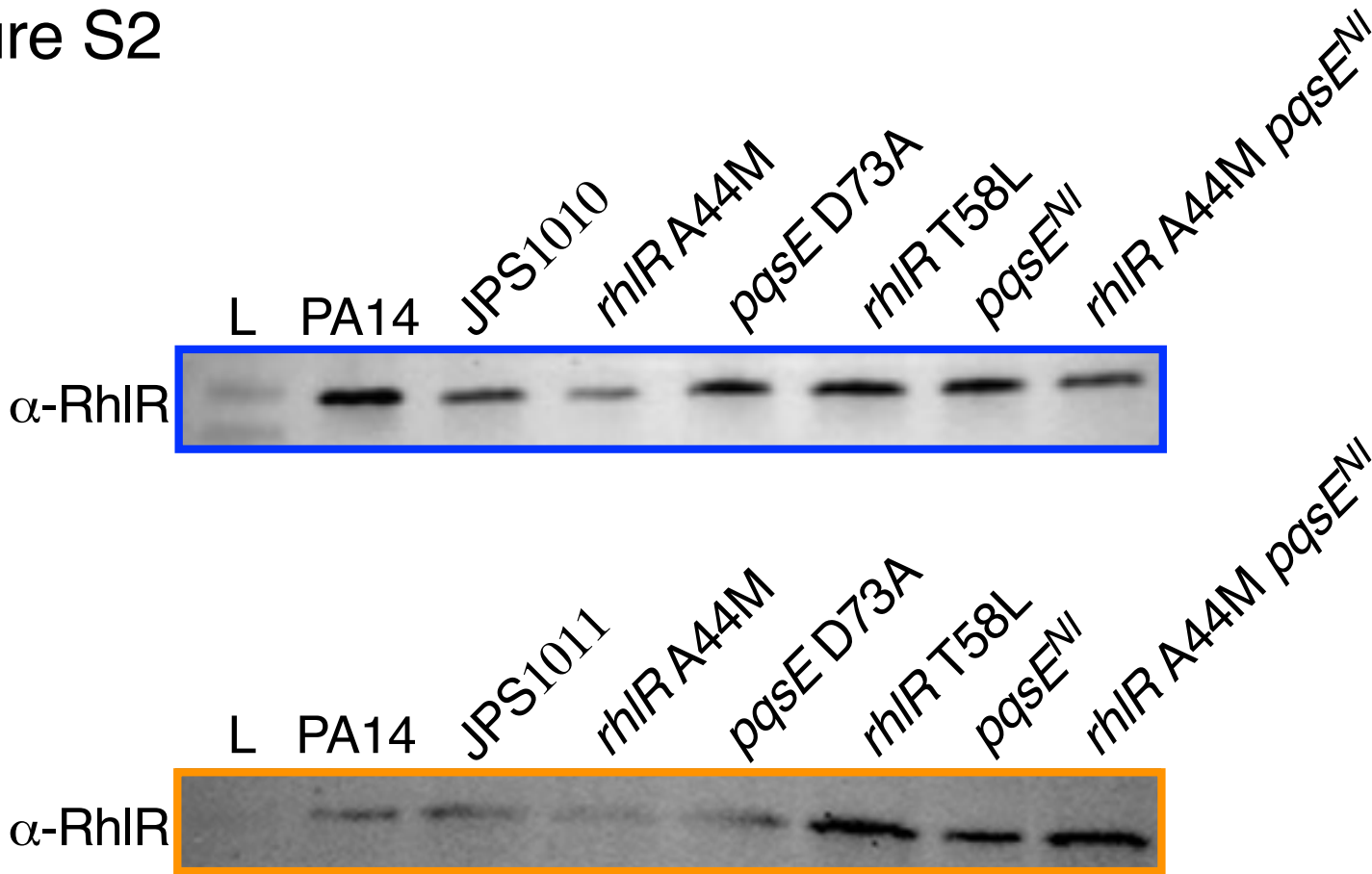

Supplement: Supplement 1 — Figure S1. Categories of mutated genes and their function. The treemap was created using the list of called variants for each isolate filtered for non-synonymous mutations and 404 genes that are QS-regulated or QS-related. Percentage indicates the ratio of called variants in the functional category. Figure S2. RhlR protein levels in clinical isolates and their mutant backgrounds. Western blot using a polyclonal antibody for RhlR against whole-cell lysates from JPS1010 (top), JPS1011 (bottom), and their respective isogenic mutants as indicated. A WT PA14 strain was used as a control. [file NIHPP2026.05.21.726854v1-supplement-1.pdf]
